# Clinician Obligations to Care for Patients Infected With Special Pathogens

**DOI:** 10.1001/jamanetworkopen.2025.54600

**Published:** 2026-01-16

**Authors:** Nina Roesner, Matthew P. Schreiber, Craig DeAtley, Shane Kappler, Maxwell Hockstein, Tani Jausurawong Wiest, Aaron Resnick, Benjamin Krohmal

**Affiliations:** 1 John J. Lynch, MD Center for Ethics, MedStar Washington Hospital Center, Washington, DC; 2 MedStar Union Memorial Hospital, Baltimore, Maryland; 3 MedStar Good Samaritan Hospital, Baltimore, Maryland; 4 Biocontainment Unit Program, MedStar Washington Hospital Center, Washington, DC; 5 Department of Critical Care, MedStar Washington Hospital Center, Washington, DC; 6 Institute for Public Health Emergency Readiness, Medstar Washington Hospital Center, Washington, DC; 7 Department of Emergency Medicine, Georgetown University School of Medicine, Washington, DC; 8 Department of Emergency Medicine, MedStar Washington Hospital Center, Washington, DC

## Abstract

This survey study assesses the views of clinical leaders of the 13 designated Regional Emerging Special Pathogen Treatment Centers in the US regarding whether they would allow clinicians to abstain from caring for infected patients.

## Introduction

Should clinicians be able to opt out of caring for patients infected with COVID-19? What if they are immunocompromised or caretakers for elderly family members? As medical professionals, hospitals, and government officials grappled with these questions during the COVID-19 pandemic, new attention was brought to one of the oldest debates in medicine: what are clinicians’ obligations when patient care entails elevated safety risk? Professional associations in the US affirm that there is a duty to treat in the face of substantial risks,^1,2^ including those associated with special pathogens, defined as highly contagious and virulent infectious agents with few or no medical countermeasures.^3^ The extent to which this duty binds individual clinicians, however, is not settled.^4^ We surveyed clinical leaders of the 13 designated Regional Emerging Special Pathogen Treatment Centers (RESPTCs), hubs for special pathogen care in the US, to assess their views on permitting clinicians to abstain from caring for infected patients.

## Methods

This survey study followed AAPOR reporting guidelines and was approved by the MedStar Health affiliated institutional review board, with a waiver of written consent. This anonymous online survey, conducted in January and February 2024, was distributed by email to all 60 clinical leaders of the 13 RESPTCs. Participants were asked whether they would support allowing clinicians to opt out of caring for an infected patient because of safety concerns, even if it required others to spend additional shifts caring for the patient, for 3 hypothetical pathogens: novel influenza comparable to past pandemic strains; viral hemorrhagic fever comparable to past outbreak strains; and disease X, a novel viral illness with unknown transmission route that is highly infectious and capable of producing severe disease in humans. To protect anonymity given the small sample, no demographic information other than professional background was collected. Details of survey administration, recruitment, and content are provided in eMethods, eAppendix 1, and eAppendix 2 in Supplement 1. Descriptive statistics were calculated using R statistical software version 4.3.1 (R Project for Statistical Computing).

## Results

Forty participants completed the survey (26 physicians and 14 nurses; 60% response rate) (Table 1). Participants had been professionally affiliated with a biocontainment unit (BCU) for a mean (SD) of 7.0 (4.2) years, and 29 (73%) had experience caring for patients infected with special pathogens or under investigation for infection within a BCU.

**Table 1.** Participants’ Professional Background and Experience

Variable	Participants, No. (%) (N = 40)
Type of health care practitioner	
Physician	26 (65)
Registered nurse	14 (35)
Primary clinical practice area^a^	
Infectious disease	18 (45)
Critical care	17 (43)
Pediatrics	7 (18)
Emergency medicine	4 (10)
General internal medicine	4 (10)
Hospitalist medicine	2 (5)
Pulmonology	1 (3)
Neurology	1 (3)
Surgery	1 (3)
Duration of professional affiliation with a clinical BCU, mean (SD), y	7.0 (4.2)
Personal experience caring for patients infected with special pathogens or person under investigation for special pathogen infections within a BCU	29 (73)

Abbreviation: BCU, biocontainment unit.

^a^ Multiple selections were allowed, so percentages do not sum to 100%.

For each pathogen, fewer than one-quarter of participants reported they would never support allowing clinicians to opt out of caring for an infected patient (novel influenza, 9 respondents [23%]; viral hemorrhagic fever, 6 respondents [15%]; disease X, 8 participants [21%]) (Table 2). The remaining participants were closely divided between those reporting they would sometimes support allowing clinicians to opt out (novel influenza, 16 respondents [40%]; viral hemorrhagic fever, 18 respondents [45%]; disease X, 15 respondents [38%]) and those reporting they would always support it (novel influenza, 15 respondents [38%]; viral hemorrhagic fever, 16 respondents [40%]; disease X, 16 respondents [41%]).

**Table 2.** Support for Allowing Clinicians to Opt Out of Caring for Patients Infected With Special Pathogens

Level of support	Participants, No. (%) (N = 40)
Novel influenza	
Always	15 (38)
Sometimes	16 (40)
Never	9 (23)
Viral hemorrhagic fever	
Always	16 (40)
Sometimes	18 (45)
Never	6 (15)
Disease X^a^	
Always	16 (41)
Sometimes	15 (38)
Never	8 (21)

^a^ Response was missing for 1 participant.

## Discussion

Although much has been written about the duty to treat, there is little discussion of the obligations of the medical profession as a whole vs those of individual clinicians. Past clinician surveys revealed divided views about individual obligations when care entails elevated risk (eg, special pathogen exposure).^5,6^ Although the results of this survey study likewise illustrate a lack of consensus among experts in special pathogen care, a large majority indicated there are situations in which they would support allowing clinicians to opt out. This is notable given that the study population’s BCU affiliation may suggest acceptance of an unusually high level of risk. Support for opting out of care may be greater among clinicians generally and warrants study. Limitations of this survey study include small sample size, potential nonresponse bias, and use of a hypothetical scenario in survey questions. Further research should also examine the reasoning underlying experts’ opinions.

## Notes

Supplement 1. eMethods. Survey administration eAppendix 1. Recruitment email eAppendix 2. Survey content

Supplement 2. Data Sharing Statement
