# Supplementary material for: Clinician Obligations to Care for Patients Infected With Special Pathogens
Source: JAMA Netw Open. 2026 Jan 16;9(1):e2554600. doi: 10.1001/jamanetworkopen.2025.54600 (PMC12811798; doi:10.1001/jamanetworkopen.2025.54600)
Supplement: Supplement 1. — eMethods. Survey administration eAppendix 1. Recruitment email eAppendix 2. Survey content [file jamanetwopen-e2554600-s001.pdf]

Supplemental Online Content

Roesner N, DeAtley C, Kappler S, et al. Clinician obligations to care for patients infected with special pathogens. *JAMA Netw Open*. 2026;9(1):e2554600.
doi:10.1001/jamanetworkopen.2025.54600

eMethods. Survey administration

eAppendix 1. Recruitment email

eAppendix 2. Survey content

This supplemental material has been provided by the authors to give readers additional information about their work.

© 2026 Roesner N et al. *JAMA Network Open*.

eMethods. Survey administration

This anonymous survey study was conducted in January and February 2024 using a self-administered online REDCap questionnaire distributed via email to all known clinical leaders of the 13 Regional Emerging Special Pathogen Treatment Centers (RESPTCs) at the time of survey administration. Initially, 59 individuals were identified using a list maintained by the National Emerging Special Pathogens Training and Education Center (NETEC) of the physician leader(s), nurse leader(s), and pediatric contact(s) for each RESPTC. Four individuals were removed from the survey distribution list after we learned that they were no longer serving as RESPTC leaders. Additionally, five individuals were identified as having been newly appointed as RESPTC leaders or inadvertently left off of the NETEC list and added to the survey distribution list. In total, the survey was distributed to 60 individuals. Recipient-specific links were sent via email followed by up to 6 reminder emails sent one week apart. Participants were informed in writing that survey responses are anonymous and deciding to proceed with the survey would indicate consent.

© 2026 Roesner N et al. *JAMA Network Open*.

eAppendix 1. Recruitment email

Dear RESPTC Leader,

You are receiving this invitation to participate in a brief online survey because you are one of around 60 clinicians listed by NETEC as a medical contact person for one of the 13 Regional Emerging Special Pathogen Treatment Centers (RESPTCs) in the United States. This survey is part of a study examining ethically complex treatment decisions for patients infected with special pathogens, and is being conducted by the John J. Lynch, MD Center for Ethics and the Biocontainment Unit at MedStar Washington Hospital Center in Washington, DC.

To generate representative results that reflect the aggregate views of RESPTC clinical leaders, we hope to obtain responses from as close as possible to 100% of this small group.

Survey completion time is approximately 10 to 15 minutes. Thank you so much for your consideration and for your time if you choose to participate.
You may open the survey in your web browser by clicking the link below:
Special Pathogen Clinical Ethics Survey of RESPTC Leaders
If the link above does not work, try copying the link below into your web browser:
https://researchdata.medstar.net/redcap/surveys/[redacted]

This link is unique to you and should not be forwarded to others.

If you believe you have received this email in error, or for more information, please contact Benjamin Krohmal at MedStar Washington Hospital Center, Department of Bioethics, at benjamin.j.krohmal@medstar.net.

© 2026 Roesner N et al. *JAMA Network Open*.

eAppendix 2. Survey content

Consent/Background

This survey is part of a research study examining ethically complex treatment decisions for patients infected with special pathogens. We are sending this survey to the nearly 60 clinical leadership contacts listed by NETEC for the 13 Regional Emerging Special Pathogen Treatment Centers (RESPTCs) in the United States.

To generate representative results that reflect the aggregate views of RESPTC clinical leaders, we hope to obtain responses from as close as possible to 100% of this small group.
Survey completion time is approximately 10 to 15 minutes. Thank you so much for your consideration and for your time if you choose to participate.
Survey responses are anonymous. While our survey software tracks which email links have been used to complete the survey, it does not connect survey responses to individuals, email addresses, or institutions.

This IRB-approved survey is being conducted by the John J. Lynch, MD Center for Ethics and the Biocontainment Unit at MedStar Washington Hospital Center in Washington, DC.

Your decision to proceed with the survey will indicate your consent to participate. For more information, you may contact Benjamin Krohmal at MedStar Washington Hospital Center, Department of Bioethics, at benjamin.j.krohmal@medstar.net.

Instructions

Please respond to the following questions based on your clinical, professional, and ethical judgment as an individual clinician, with the understanding that these judgments may not necessarily reflect policies or practices at your institution.

In the scenarios described below, please select the answer choices that most closely align with your judgment given the limited information provided. Assume standard availability of PPE and other medical equipment and resources.

Definitions

Please use the following definitions to answer the survey questions:
- "Novel Influenza": a newly identified strain of influenza that appears to have a route of transmission, virulence, and infectiousness comparable to strains involved in major 20th and 21st Century influenza pandemics
- "Viral Hemorrhagic Fever": a strain of viral hemorrhagic fever that appears to have a route of transmission, virulence, and infectiousness comparable to viral hemorrhagic fever strains involved in periodic outbreaks over the previous decade
- "Disease X": a novel viral illness with unknown route of transmission that is highly infectious and capable of producing severe disease in humans

Relevant questions

**In general, I would support allowing clinicians to opt out of caring for a patient infected with the following special pathogen due to safety concerns, even if this might require others to spend additional shifts caring for the patient:**			
	**Always**	**Sometimes**	**Never**
Novel Influenza			
Viral Hemorrhagic Fever			
Disease X			

What type of health care provider are you	Physician<br>Nurse Practitioner<br>Physician Assistant<br>Registered Nurse

© 2026 Roesner N et al. *JAMA Network Open*.

	Pharmacist<br>Other (specify:)

What is your primary clinical practice area(s)? (select all that apply)	Anesthesiology<br>Cardiology<br>Critical care<br>Emergency medicine<br>General internal medicine<br>Geriatrics<br>Hospitalist medicine<br>Infectious disease<br>Nephrology<br>Palliative care<br>Pediatrics<br>Pulmonology<br>Neurology<br>OB/Gyn<br>Orthopedics<br>Psychiatry<br>Physical medicine & rehabilitation<br>Radiology<br>Surgery<br>Other (please specify below)

For approximately how many years have you been professionally affiliated with a clinical biocontainment unit?	

Do you have personal experience caring for patients infected with special pathogens or PUIs within a biocontainment unit?	No<br>Yes (please specify which special pathogens below:)

© 2026 Roesner N et al. *JAMA Network Open*.
