# Supplementary material for: Clinician Obligations to Care for Patients Infected With Special Pathogens
Source: JAMA Netw Open. 2026 Jan 16;9(1):e2554600. doi: 10.1001/jamanetworkopen.2025.54600 (PMC12811798; doi:10.1001/jamanetworkopen.2025.54600)
Supplement: Supplement 2. — Data Sharing Statement [file jamanetwopen-e2554600-s002.pdf]

Data Sharing Statement

Roesner. Clinician Obligations to Care for Patients Infected With Special Pathogens. *JAMA Netw Open*. Published January 16, 2026. doi:10.1001/jamanetworkopen.2025.54600

Data
Data available: No
